# Children Immunization App (CIMA): A Non-randomized Controlled Trial Among Syrian Refugees in Zaatari Camp, Jordan

**DOI:** 10.1007/s10935-023-00721-7

**Published:** 2023-01-17

**Authors:** Soha El-Halabi, Yousef S. Khader, Mohammad Abu Khdeir, Claudia Hanson, Tobias Alfvén, Ziad El-Khatib

**Affiliations:** 1 Department of Global Public Health, Karolinska Institutet, Stockholm, Sweden; 2 Department of Community Medicine, Public Health and Family Medicine, Jordan University of Science & Technology (JUST), Irbid, Jordan; 3 Ministry of Health, Amman, Jordan

**Keywords:** mHealth, Refugees, Vaccines, Jordan, Syria

## Abstract

Approximately 20 million children are not vaccinated, especially among refugees. There is a growing access to smartphones, among refugees, which can help in improving their vaccination. We assessed the impact of an app for the vaccination follow-up visit among refugees in Jordan. We developed an app and tested it through a non-randomized trial at the Zaatari refugees camp in Jordan. The study was conducted during March – December 2019 at three vaccination clinics inside the camp. The study included two study groups (intervention and control groups) for refugees living at the camp. The intervention group included parents who own an Android smartphone and have one newborn that require between one and four first vaccination doses and they accepted to participate in the study, during their regular visit to the vaccination clinics. The control group was for the usual care. We compared both study groups for returning back to one follow-up visit, using Kaplan-Meier survival analysis. We recruited 936 babies (*n* = 471; 50.3% in the intervention group, both study groups were similar at baseline). The majority of mothers were literate (94.2%) with a median age of 24. The majority of the babies had a vaccination card (*n* = 878, 94%). One quarter (26%) of mother-babies pairs of the intervention group came back within one week (versus 22% for control group); When it comes to lost-follow-up, 22% and 28% did not have a history of returning back (intervention and control groups respectively, *p* = 0.06) (Relative risk reduction: 19%). The Kaplan-Meier Survival Analysis showed a statistically significant progressive reduction in the duration of coming back late for the follow-up vaccine visit. We tested a vaccination app for the first time, in a refugee population setting. The app can be used as a reminder for parents to come back on time for their children’s vaccine follow-up visits.

## Introduction

Globally, children immunization is a life saving intervention, especially in fragile contexts where there is an increased risk for acquiring infectious diseases (UNICEF, 2018). Yet, over 20 million children are still unvaccinated, therefore they are at risk of acquiring Vaccine Preventable Diseases (VPDs) and, potentially, unnecessary deaths (World, 2015). The refugees are at a high risk of VPD, especially as half of them are below 18 years of age (El-Khatib, 2013). In conflict settings, such as Syria, the total number of measles cases also increased from 13 to 740 in late 2011 and early 2013 (approximately an increase of 57 folds in less than 12 months) (World, 2016).

Over three million children have fled out of Syria, since 2011, to the Middle East and Europe (EuroStat, 2015) and they were exposed to several risks on their way, including passing or living in countries with low vaccination coverage (World, 2015). For example, the proportion of children that are vaccinated, among Syrian refugees, in Jordan and Lebanon is 25% and 13%, respectively (Roberton et al., 2017). This increases the risk of outbreaks in these countries, particularly among individuals who have not been vaccinated at all or completed vaccination schedules (European, 2014). Also, the estimated hospitalization cost may reach US$25,000 per case (Walker et al. 2015). Therefore, the influx of children without clear immunization records creates a challenge for health providers to maintain herd immunity for the unvaccinated children (World, 2015; El-Khatib, 2013; European, 2014; Lam, 2015; Sharara et al., 2014). The vaccination rate in the hosting country is also considered low, for example in Jordan, vaccination rate among the general population is estimated to be < 50% (United 2018). Given the risk to unvaccinated children both in the refugee populations and the general population, it is vital to provide support to health professionals to monitor and increase the rate of vaccination among refugee children. The challenge for monitoring vaccinations among refugees is compounded by the use of the yellow vaccination card, which are easily lost or not brought to medical consultations. The mobile phone-based application Health (mHealth) could be used as an alternative for paper-based vaccination records, and a smartphone application (app) could present advantages in empowering parents by informing them of vaccination schedules and dates and allow them to monitor vaccination coverage on their own.

There is a growing evidence on the effectiveness of smartphone apps of reminders interventions, e.g. automated telephone reminders, on improving vaccine uptake and series completion (Atkinson et al., 2019), though there remains a gap in evidence of effectiveness among refugees. Smartphones provide novel approach to solve problems with data registration, transmission and storage (World, 2011; El-Khatib, 2018; El-Halabi, 2018; El-Khatib, 2018). Refugees use smartphones as a survival kit, to connect with their social networks and to search for information about their host countries (Walker et al., 2015). However, there remains a gap of well-conducted evaluations of mHealth interventions among refugee populations, particularly with respect to maternal and child health. According to our knowledge, no study has used an app to support refugees’ population in recording their vaccination records and to provide them an automated reminder for the vaccination visits.

We have implemented an integrated app intervention in collaboration with the Jordan Ministry of Health, Jordan University of Science and Technology (JUST), United Nations Children’s Fund (UNICEF), United Nations High Commissioner for Refugees (UNHCR), and the local health service delivery partners in the Zaatari camp. This study aimed to assess the impact of the automated reminder for the vaccination visits delivered via an app, called the Children Immunization App (CIMA) (Khader, 2019; El-Khatib, 2020), on the likelihood of returning of children to their vaccines follow-up visit within 0–7 days of their scheduled appointment day at the Zaatari camp in Jordan.

## Methods

### Study Setting

The study was conducted at the Zaatari camp in Jordan, which is considered one of the largest hosting camps for refugees, in Jordan and the Middle East. The camp was first opened in 2012, to host the Syrian refugees, fleeing the Syrian civil war. The camp population is estimated to be hosting 80,000 refugees (area size of 5.3 Km^2^, approximately 15,000 persons per Km^2^), where approximately 20% (*n* = 20,000) of them are under five years of age. The Zaatari camp is located in Northern part of Jordan, near the southern borders of Syria. The Zaatari camp has a basic infrastructure, where all households are made out of containers and there is an installed system for water, sewage and electricity that is relies on both of solar panels and government electricity network. There is a total of eight clinics providing vaccine services inside the camp. The Jordanian Ministry of Health provides a full subsidy of the vaccines, as well as it manages the vaccination supply storage and distribution inside the Zaatari camp.

### Study Design and Study Participants Recruitment

This was a non-randomized controlled trial to evaluate the effectiveness of using an app to record the vaccination schedule, including reminders for parents, on increasing immunization coverage of Syrian children at the Zaatari refugees’ camp in Jordan. The study, including study participants recruitment, was conducted during the period of March through December 2019. The clinics, were located inside the camp, and they provide vaccination services for the children. We choose the clinics to be far from each other to avoid contamination effect (i.e. the clinic site would fall under the control or the intervention study group). The study was announced through posters in Arabic, in the clinics. Clinicians and social workers also informed the residents of the camp about the study. Parents interested in joining the study were fully informed about the study details. Parents who provided their informed consent were included in the study. Three vaccination clinics were included, where two clinics were under the intervention study, and one clinic was under the control study for the regular care. The intervention study arm provided the CIMA app in addition to the regular care, while the control study arm received the regular care (using the vaccination card) in addition to the usual information on the benefits of vaccination. We recruited a total of 936 children where 50.3% (*n* = 471) in the intervention group (Khader, 2019).

### CIMA App Description

During the period of August 2018 through January 2019, the CIMA app has been designed, and developed in English and Arabic languages by El-Khatib 2020. Also we have conducted an in-house testing for the technical functions of the app (e.g. to download the app and test its functions on fictional accounts and on different smartphone devices).The CIMA app included four layers: (i) Health promotion messages for the benefits of vaccination that show up on the main page; (ii) Storing the post of vaccination for each child, according to the vaccination schedule of the Jordan Ministry of Health, on the parents’ smartphones in Arabic and in English languages (in an interchangeable fashion); (iii) Displaying the vaccination schedule, for each child, using green, orange and red colors depending on vaccination status if it was received, due or overdue respectively; (iv) Appointment reminder was displayed on the users phones at four different time-points prior the vaccination schedule (one week, three days, 1 day and the morning of the appointment). Then the users received two notifications in the coming days of the scheduled vaccine in case of missing the appointment. Participants downloaded the CIMA app, at no cost, on their personal devices (Android only) with the help of the study staff (the link was invisible to public access during the study recruitment period, to avoid any contamination effect with the control study arm).

### Study Groups

#### Intervention

The parents were recruited to the study by trained volunteers at the local vaccination clinic, providing vaccinations, around the Zaatari camp. The app offered the following functions: (a) Allowed storing Jordanian vaccination records, per child, on the parents’ smartphones in Arabic and in English languages (in an interchangeable fashion); (b) Every vaccination record had a set of automated reminders prior the appointment of each child. The appointment reminder was displayed on the users’ phones at four different time points before the vaccination schedule (one week, three days, and one day and the morning of the appointment). Thereafter, the users received two notifications in the coming days of the scheduled vaccine in case of missing the appointment (at one and two weeks time); and (c) Summarized the immunization records in form of “due”, “taken” or “overdue” appointments, labeled in orange, green and red respectively. The inclusion criteria of the study were (i) having at least one child age 0–5 years of age; (ii) being a local resident of the camp and (iii) having an Android smartphone that can allow CIMA app installation.

#### Control

In the control group, the clinic nurse explained the study to the parents (same inclusion and exclusion criteria as the intervention group). After giving the consent to participate in the study, the nurse interviewed the parents for the baseline questionnaire.

## Assessments

Baseline assessment included socio-demographic data, any prior vaccine history and eHealth literacy (Schnall et al., 2016). Participants, in both study arms, were monitored for their follow-up visits to the clinic for the vaccination doses. The vaccination cards of both study arms were marked as “intervention” or “control” arm, so the clinic nurses could notify the field workers about the follow-up visits. For the study outcome measures, we measured any differences in the proportion of coming back on time, defined coming back within 14 days post the next vaccination visit.

### Statistical Analysis

The analysis was done using a set of steps. In Step 1, we described the baseline characteristics of the participants and conducted a comparison between the intervention and the control groups (for all study participants and for the ones that did not come back during the study period) using independent t-test for continuous variables and Chi-square test for categorical variables. In Step 2, we calculated relative risk reduction; and finally, in Step 3, we conducted Kaplan-Meier survival analysis to further contrast the difference in the proportion of defaulters between the intervention and comparison groups, using the outcome of coming back to the clinic appointment during the study period. All data analysis was carried using Stata/MP 14.0.

### Ethical Considerations

This study has been reviewed and approved by the Institutional Review Board of the Jordan University of Science and Technology (JUST) (Reference# 14/112/2017, date 14/1/2018). Also, the project proposal has been endorsed by the Minister of Health in Jordan, UNICEF-Jordan and we obtained the security clearance from the office of the United Nations High Commissioner for Refugees (UNIHCR) that has the full mandate of protecting the Zaatari camp. Due to the vulnerability of the refugees and the context of the camp, all participants were invited to participate on a voluntary basis. Survey data was collected, at baseline, and follow-up visits dates were recorded using study ID numbers. No personal information was stored. The study participants had their full right to cancel their participation in the study, including closing their study file, at any time during the study period.

## Study Funding

This study has been funded by (i) Grand Challenges Canada, which is funded by the Government of Canada and is dedicated to supporting Bold Ideas with Big Impact ® (GCC grant ID: R-ST-POC-1807-12490); (ii) The Karolinska Institutet foundations and funds – Karolinska Institutet research foundation grants.

## Results

### General Characteristics of the Study Population

A total of 936 babies were recruited in this study, where half of them (*n* = 471/926; 50.3%) were in the intervention study group (Table 1). Overall, the average age of the mothers was 24.3 and 30.5 years for the fathers. The majority of the mothers and fathers reported that they have been to school (87.1% and 86.3% respectively). The average number of children per family was three and less than half of the babies were girls (*n* = 408/936; 43.8%). Most of the study participants reported mothers as the main decision makers about the vaccination of the children (*n* = 728/936; 77.8%).

**Table 1:** Characteristics of the study participants in the control and intervention study groups

Characteristics	All		Intervention		Control		*p* value
	*N* = 936	(%)	*N* = 471	(%)	*N* = 465	(%)	
*Age*							
Mothers age – Median (IQR)*	24.3	(20.3 ; 30.3)	23.9	(20.1; 30.3)	24.9	(20.6; 30.5)	0.12
Mothers age – Mean (*SD*)	25	(6.7)	25.5	(6.8)	26.1	(6.6)	
Fathers age – Median (IQR)	29.2	(24.6; 35,2)	29.0	(24.4; 35.3)	29.3	(24.9; 34.9)	0.75
Fathers age – Mean (*SD*)**	30.5	(7.3)	29.0	(7.5)	30.5	(7.1)	
*Educational level*							
Mothers							
Have not been to school	57	(6.1%)	25	(5.3%)	32	(6.9%)	
Have been to school	815	(87.1%)	404	(85.8%)	411	(88.4%)	
Post school technical or university education	64	(6.8%)	42	(8.9%)	22	(4.7%)	**0.03**
Fathers							
Have not been to school	30	(3.4%)	14	(3.2%)	16	(3.5%)	
Have been to school	770	(86.3%)	392	(89.1%)	378	(83.6%)	
Post school technical or university education	92	(10.3%)	34	(7.7%)	58	(12.8%)	**0.04**
Total number of children per parent – Mean (*SD*)	3	1.9	3	1.9	3.4	1.9	**< 0.01**
*Gender of the child enrolled in the study*							
Girls	408	(43.8%)	275	(58.8%)	249	(53.7%)	
Boys	524	(56.2%)	193	(41.2%)	215	46.3%)	0.12
*Decision maker for vaccination in household*							
Mothers	728	(77.8%)	319	(67.7%)	409	(88.0%)	
Fathers	208	(22.2%)	152	(32.3%)	56	(12.0%)	**< 0.01**
*Came back to clinic*							
< 7 days	212	(22.7%)	116	(24.6%)	96	(20.7%)	
7–30 days	266	(28.4%)	122	(25.9%)	144	(31.0%)	
> 30 days	222	(23.7%)	127	(27.0%)	95	(20.5%)	
Did not come back during the study period***	235	(25.1%)	106	(22.5%)	129	(27.8%)	**0.01**

*p* values < 0.05 are given in bold

*IQR: Inter-quartile range; **SD: Standards deviation; ***The parents that never came back could have moved outside the camp, but we had no possibility to trace them

In the intervention study group, the proportion of parents that have been to a post school technical or university education was higher for mothers, and lower for fathers, in comparison to the control group. A higher percentage of fathers decided about the children’s vaccination level in the intervention group. Finally, the percentage of babies that came back to the follow-up vaccination visit, within 7 days of the appointment, was higher for the intervention group (Table 1). When it comes to the characteristics of the study participants that did not come back to the clinics, during the study period, there was no statistical difference between them and the group that came back (in each of the intervention and control groups respectively) (Table 2).

**Table 2:** The comparison of the characteristics of children that came back and never came back in each of the intervention and controls study groups

	Intervention		*p*-value	Control		*p*-value
	Came back	Never came back		Came back	Never came back	
	*N* = 508	*N* = 168		*N* = 192	*N* = 67	
*Age*						
Mothers age - Median (IQR)*	23.9 (20.3; 30.4)	23.8 (19.5; 30.0)	0.52	25.6 (21.2; 30.6)	25.3 (20.2; 30.2)	0.73
Mothers age – Mean (*SD*)	25.7 (6.9)	25.2 (6.5)		26.5 (6.3)	26.4 (6.8)	
Fathers age - Median (IQR)	28.8 (24.6; 35.3)	27.7 (24.3; 33.7)	0.14	30.2 (25.5; 35.6)	29.4 (24.3; 35.7)	0.63
Fathers age - Mean (*SD*)**	30.5 (7.1)	29.7 (7.6)		31.2 (7.5)	30.7 (7.3)	
*Educational level*						
Mothers						
Have not been to school	28 (5.5%)	13 (7.7%)		9 (4.7%)	7 (10.4%)	
Have been to school	442 (87.0%)	144 (85.7%)		172 (89.6%)	56 (83.6%)	
Post school technical or university education	38 (7.5%)	11 (6.6%)	0.55	11 (5.7%)	4 (6.0%)	0.24
Fathers						
Have not been to school	18 (3.7%)	10 (6.5%)		1 (0.5%)	1 (1.6%)	
Have been to school	418 (85.0%)	120 (78.4%)		177 (95.7%)	54 (88.5%)	
Post school technical or university education	56 (11.4%)	23 (15.0%)	0.13	7 (3.8%)	6 (9.8%)	0.13
Total number of children per parent - Mean (*SD*)	3.2 (1.9)	3.0 (1.8)	0.22	3.5 (2.0)	3.5 (2.0)	0.97
*Gender of the child enrolled in the study*						
Girls	221 (43.8%)	72 (42.9%)		91 (47.6%)	23 (34.3%)	
Boys	284 (56.2%)	96 (57.1%)	0.84	100 (52.4%)	44 (65.7%)	0.06
*Decision maker for vaccination in household*						
Mothers	357 (89.5%)	127 (90.7%)		182 (97.8%)	61 (98.4%)	
Fathers	42 (10.5%)	13 (9.3%)	0.68	4 (2.1%)	1 (1.6%)	0.79

*IQR: Inter-quartile range; **SD: Standards deviation; ***The parents that never came back could have moved outside the camp, but we had no possibility to trace them.

## Vaccines Appointments

Of the total 936 babies, 212 (22.7%) babies came back to their vaccine follow-up visit within 0–7 days of their scheduled appointment day. In the intervention group, 24.6% (*n* = 116/471) of babies came back on time, versus 20.7% (*n* = 96/465) of babies in the control group (*p* = 0.01). Babies who never came back, during the study period, were 22.5% (*n* = 106/471) and 27.8% (*n* = 129/465) in the intervention and control groups, respectively (Table 1).

**Fig. 1:**
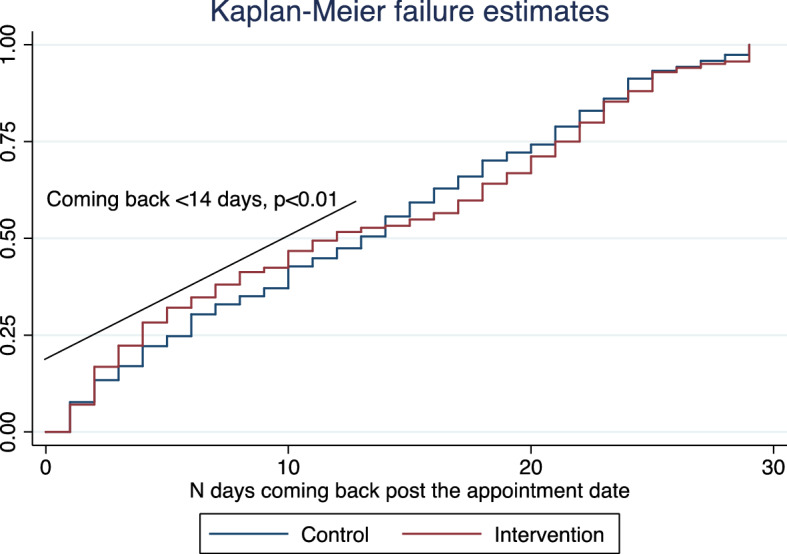
Kaplan-Maier survival analysis for coming back on time for the vaccination appointment

The relative risk reduction rate in the chance to come back late for the vaccination appointment was 19% for the intervention group (Table 3). Analyzing the risk of coming late to vaccine appointment, using Kaplan Meier survival analysis, showed a statistically significant reduction in coming back, within 0–14 days, within the vaccine appointment period (*p* < 0.01) (Fig. 1).

**Table 3:** Description of the relative risk reduction for the intervention group

Characteristic	%
Experimental* event rate (EER)	26.1
Control event rate (CER)	21.9
Relative risk reduction (RRR) – Intervention group = |EER - CER| / CER	19.0

*Experimental = Intervention group.

## Discussion and Final Remarks

The objective of the pilot project was to test the effectiveness of using an automated reminder, integrated in an app, for the vaccination appointment in a refugee population (Khader, 2019; El-Khatib, 2021). In comparison with the card-based vaccination appointments, the proportion of babies that came back on time was higher in the intervention group. The app provided three automated reminders prior the appointment and two automated reminders, in the case of missing the appointment. The parents of this cohort, reported, in a different study, that the reminders were helpful for them, in addition to the health educational messages around vaccines and their importance (Khader et al., 2022). The refugee population is a vulnerable group, especially when it comes to adjusting to a new setting and with all certainties regarding their settlement (Kiselev et al., 2020). Also, parents with low health literacy about vaccination benefit are reported to delay their children to receive all of their vaccines (Debela, 2022). Therefore, we tried to address these needs by providing information about the benefits of vaccines, in form of visual information and simple text, as developed by experts at UNICEF and the World Health Organization (Khader et al., 2022). We observed a slight difference in the characteristics of the parents’ education level, between both study groups; however, all residents of the Zaatari camp come from the same region in Southern Syria (however we cannot confirm it in our study as we did not ask them about their area of origin in Syria).

During the recruitment of the study, the babies were mainly accompanied by their mothers; but we observed, in the intervention group, that mothers would request the consent of the fathers too so they can be included in the study. Then later on, the nurses observed that the fathers became more engaged in the vaccination process of their children. Few anecdotes included that the fathers felt that the children vaccination must be an important topic if it is recorded on an app. Also anecdotes included that women felt empowered regarding the importance of the vaccination of their children, because they are recorded on an app.

Additionally, the proportion of the babies that never came back to the clinic was lower in the intervention group. However, when we compared the characteristics of the study parents that never came back, we could not identify a statistical difference between the two study groups.

Smartphones-based vaccination apps have been pilot tested in several contexts in the past decade. A systematic review, by Atkinson et al., report a total of 13 empirical studies where they compare digital to non-digital reminders for the completion of vaccination for children age ≤18 among the general population (i.e. none was done among refugees) (Atkinson et al., 2019). It is relatively a field with a short-term evidence with high heterogeneity and where further evaluation is needed (Atkinson et al., 2019). Yet, our pilot, in Zaatari camp, was further evaluated by the community and showed a high sense of affinity. The parents reported a high level of trust in the app due to their trust in the clinics and the strong commitment of the Jordanian Ministry of Health to provide vaccines in the camp (Khader et al., 2022).

The Zaatari camp is located near the Syrian borders, therefore it is a vulnerable area for outbreaks and communicable diseases. The Jordanian Ministry of Health considers this area to be of a high importance for outbreaks prevention, by ensuring the children are fully vaccinated inside the Zaatari camp. In a separate study, we conducted the feasibility of scaling the CIMA app and it is estimated to be 0.25$ per child (Thomas, 2022). According to our knowledge, this was the first time a vaccination schedule app was tested in a refugee camp. However, we should mention a few limitations. The invitation for the study was done using a passive approach (i.e. a non active recruitment method), where we informed parents through posters; therefore it was not possible to identify the total number of parents that were qualified for the study and not interested in joining the study and we could not calculate the acceptance rate. The project was of a limited one-time appointment, and we could not conduct a follow-up on the reason why the babies never came back to their vaccination appointments. We did not assess the effectiveness of the reminders on more than one-time appointment. We did not include a qualitative evaluation of the parents’ perception of the app, due to the observed anecdotes about how parents felt engaged and have a responsibility towards their children vaccination appointments.

The vaccination app can be used as a reminder for parents to come back on time for their children’s vaccine follow-up visits.
